# 

**Published:** 1971-04

**Authors:** 


					t> 'P,.
^ Y
0CT|2K/1 V
nn, oi M^aioino .v-';-
?
- -* ? ' ^ ^ g ""?* '-': .
MEDICO
CHIRURGICAL
JOURNAL
w. ?"?w.-rrr:
juun IVML
. :
APRIL 1971
VOL. 86 (ii) No. 318

				

